# Impact of Oil Bodies on Structure, Rheology and Function of Acid-Mediated Soy Protein Isolate Gels

**DOI:** 10.3390/foods13091289

**Published:** 2024-04-23

**Authors:** Songbin Liu, Zhihao Zhao, Pengfei Zhou, Yuanyuan Deng, Guang Liu, Ping Li, Jiarui Zeng, Yi Zhang, Mingwei Zhang

**Affiliations:** 1 College of Food Science, Fujian Agriculture and Forestry University, Fuzhou 350002, China; gmp.gap.iso@gmail.com; 2 Sericultural & Agri-Food Research Institute Guangdong Academy of Agricultural Sciences, Key Laboratory of Functional Foods, Ministry of Agriculture and Rural Affairs, Guangdong Key Laboratory of Agricultural Products Processing, Guangzhou 510610, China; zhaozhihao1991@163.com (Z.Z.); pfzhougz@foxmail.com (P.Z.); yuanyuan_deng@yeah.net (Y.D.); liuguang@gdaas.cn (G.L.); liping2019@gdaas.cn (P.L.); zengjiarui@hotmail.com (J.Z.)

**Keywords:** acid-mediation, emulsion-filled gels, microstructure, oil bodies, soy protein isolate

## Abstract

Oil bodies (OBs) are naturally occurring pre-emulsified oil droplets that have broad application prospects in emulsions and gels. The main purpose of this research was to examine the impact of the OB content on the structure and functional aspects of acid-mediated soy protein isolate (SPI) gel filled with OBs. The results indicated that the peanut oil body (POBs) content significantly affected the water holding capacity of the gel. The rheological and textural analyses showed that POBs reduced the gel strength and hardness. The scanning electron and confocal laser scanning microscopy analyses revealed that POBs aggregated during gel formation and reduced the gel network density. The Fourier transform infrared spectrum (FTIR) analysis demonstrated that POBs participated in protein gels through hydrogen bonds, steric hindrance and hydrophobic interactions. Therefore, OBs served as inactive filler in the acid-mediated protein gel, replaced traditional oils and provided alternative ingredients for the development of new emulsion-filled gels.

## 1. Introduction

An emulsion-filled gel is an emulsion with a gel-like network structure and solid mechanical properties [1,2]. Numerous food items, including yogurt, cheese and processed meat, can be classified as emulsion-filled gels [3]. Obtaining an emulsion-filled gel typically requires the initial preparation of the emulsion. However, as thermodynamically unstable systems, emulsions are often disrupted by coagulation, coalescence or phase separation. This can impact their physicochemical properties and sensory attributes [4]. Therefore, while preparing an emulsion-filled gel, it is common to add food emulsifiers to facilitate forming and stabilizing the emulsion [5]. However, recent studies have identified potential safety risks associated with food emulsifiers. Long-term ingestion of polysorbate-80 and carboxymethyl cellulose has been demonstrated to disrupt the intestinal microbiota in mice, leading to colitis and metabolic syndrome [6]. Randomized controlled trials have also confirmed that consuming 15 g of carboxymethyl cellulose per day for 11 consecutive days can disrupt the gut microbiota and metabolism in healthy human adults, promoting the onset of chronic inflammatory diseases [7]. Therefore, reducing, or even entirely eliminating, the use of emulsifiers during food production is very important.

Oil bodies (OBs) are cellular organelles that reposit lipids in plant seeds [8]. They are composed of a central triglyceride core enveloped by a phospholipid monolayer with embedded OB proteins [9]. This unique structure endows OBs with excellent physicochemical stability [10,11]. OBs can be rapidly separated using water extraction methods based on density differences. The obtained OBs are in a naturally emulsified lipid form, capable of dispersing into the aqueous phase to form OB emulsions without the help of emulsifiers [12]. Furthermore, the lipids within OBs are not extracted or refined, thus the loss of active ingredients during processing is avoided [13]. OBs possess advantages such as pre-emulsification, convenient extraction and nutritional abundance, offering broad prospects for their use in emulsified foods. Currently, there are studies exploring the application of OBs in products such as yogurt, fresh cream, hazelnut spread, fat substitutes and active ingredient delivery systems [14,15].

Soy protein isolate (SPI) is extensively utilized in the food industry due to its exceptional gelation properties, low cost, and high nutritional value [16,17]. SPI gel can be obtained by inducing protein denaturation using heat and subsequently adding acid or salt for gelation [18]. The SPI gel containing oil droplets is believed to be a protein gel matrix with embedded oil droplets, where both components interact and contribute to forming a stable gel network [19,20]. During this process, the type and amount of oil significantly impact the gelation and network structure formation of acid-induced SPI emulsion-filled gels [21,22]. Due to their inherent emulsification properties, OBs have the potential to substitute conventional oil or fat droplets in emulsion-filled gels [12]. Despite the existing studies on OB-filled emulsion gels and their properties, limited information is available regarding the impact of OBs on the textural properties and microstructure of acid-induced SPI gels.

Hence, the objective of this research is to examine the influence of peanut oil body (POBs) content on the rheological and textural characteristics of Glucono-δ-lactone-induced SPI gel. The dispersion of POBs in the protein gel matrix and the microstructure of the gel were analyzed through confocal laser scanning microscopy (CLSM) and scanning electron microscopy (SEM). This exploration aimed to enhance the comprehension of the impact of OBs on the physicochemical characteristics of acid-induced SPI gels and provide valuable perspectives for utilizing OBs in gel-state emulsified food products.

## 2. Materials and Methods

### 2.1. Materials

Peanut seeds (Luhua 11 variety) were purchased from a local supermarket. Glucono-δ-lactone (GDL) was obtained from Shanghai Macklin Biochemical Co., (Shanghai, China), while other chemicals of analytical reagent grade were obtained from Sinopharm Chemical Reagent Co., Ltd. (Shanghai, China). Distilled water is prepared in the laboratory.

### 2.2. SPI Dispersion Preparation

SPI was dissolved in deionized water at 25 °C using a magnetic stirrer for 4 h to prepare a stock solution containing a 5% (*w*/*w*) SPI concentration. The SPI stock solution underwent a thermal treatment in a water bath (90 °C for 30 min), ensuring the complete denaturation of proteins. After cooling to 25 °C, the solution was put in a refrigerator and stored at 4 °C.

### 2.3. Peanut Oil Body Extraction

The preparation of the peanut oil body was conducted using a modified version of the method described by Chen et al. [23]. After triple rinsing peanuts using deionized water, they were immersed in deionized water at a 1:6 (*w*/*v*) ratio and kept at 4 °C for a duration of 10 h. They were then homogenized in a high-speed homogenizer for 2 min, followed by filtration through four layers of gauze. The peanut milk subsequently underwent a centrifugation at 4 °C (8000× *g*, 20 min) to collect the upper layer of POBs. The POBs were refrigerated at 4 °C for future use.

### 2.4. Mixed Gel Solution Preparation

Using the SPI solution without POBs as a control, POBs of different masses were added to the SPI solution and magnetically stirred for 30 min to obtain a mixed gel solution with POB concentrations of 10%, 20%, 30%, 40% and 50% (*w*/*w*). These mixed gel solutions containing POBs were denoted as OB emulsions.

### 2.5. Preparation of Acid-Induced OB Emulsion-Filled SPI Gel

1% GDL was added to the mixed gel solution and gently dispersed by mixing on a magnetic stirrer for 5 min. The mixture was heated in a water bath at 70 °C for 30 min. The sample was refrigerated at 4 °C overnight after it had cooled to room temperature. The mixed gel solutions containing POBs were denoted as OB emulsion-filled gels.

### 2.6. Mixed Gel Solution Characteristics

#### 2.6.1. Measurement of Gel Solution Particle Size and ζ-Potential

The particle size of the gel solution was determined using a Mastersizer 3000 laser diffraction particle size analyzer (Malvern Instruments, Worcestershire, UK). The emulsion droplets had a refractive index of 1.46, while the aqueous dispersion medium exhibited a refractive index of 1.33. The ζ-potential measurements were performed utilizing a Malvern Zetasizer Nano ZSE (Worcestershire, UK). Prior to measuring the ζ-potential, each sample was diluted 500× in deionized water.

#### 2.6.2. Measurement of Physical Stability

The turbidity stability index (TSI) was determined using a Turbiscan Lab Expert (Formulaction, Toulouse, France) to characterize the physical stability of the gel solution. The testing conditions included temperature control at (25 ± 0.5) °C, a sample volume of 20 mL, a scanning frequency of 1 scan/min and a scanning duration of 30 min.

### 2.7. Functional Characteristics

#### 2.7.1. Water Holding Capacity

An appropriate amount of gel was added into a tube for centrifugation. The combined weight was accurately measured and then the sample was centrifuged at 10,000× *g* for 10 min. Water was then removed by blotting with filter paper. The combined weight of the centrifuge tube and the sample was subsequently remeasured.

The water holding capacity (WHC) was calculated as below:

WHC = (W2 − W0)/(W1 − W0) × 100%(1)

where:

W2 represents the combined weight of the centrifuged sample (g),
W0 represents the weight of the empty centrifuge tube (g),
W1 represents the combined weight of the sample before centrifugation (g).

#### 2.7.2. Measurement of Textural Characteristics

The gel solution was formed into a gel sample with a height of 2.5 cm and a diameter of 1.5 cm. The texture profile analysis (TPA) was conducted using a TA-XT plus physical property analyzer equipped with a P/0.5R cylindrical test probe (Stable Micro Systems Ltd., Godalming, UK). The pre-test speed, test speed and post-test speed were set at 0.5 mm/s. The deformation amount was 40%. The time interval was 5 s and the trigger force was 5 g.

#### 2.7.3. Dynamic Rheological Analysis

Rheological measurements of the gel were tested using an AR-1500EX shear rheometer (TA Instruments, New Castle, DE, USA). The 40 mm parallel plate was selected with a test gap of 1 mm. A 0.1–10 Hz/s frequency sweep testing of the gel was performed at 25 °C and a constant strain of 1% (in the linear viscoelastic region), while the apparent viscosity was measured under a shear rate ranging from 0.1 to 10 S^−1^. 

The temperature sweep was performed by immediately transferring the gel solution after adding GDL to button plate, using low-viscosity silicone oil to moderate moisture loss during the gelation process. The gel was rapidly heated from 25 to 70 °C at 10 °C per minute, while subjected to a 1 Hz oscillation at a 1% strain and then incubated at 70 °C for 30 min. The samples were subsequently cooled to 25 °C at 10 °C per minute. The storage modulus (G′) and loss modulus (G″) were recorded.

### 2.8. Microstructures

#### 2.8.1. Confocal Laser Scanning Microscopy Analysis

The fluorescent micrographs of the gels were obtained by an LSM-710 confocal laser scanning microscope (Carl Zeiss, Oberkochen, Germany). The staining of protein and lipid constituents was performed using Nile Blue and Nile Red, respectively. After staining, the sample microstructure was observed under the excitation of a wavelength of 633 nm for Nile Blue and 488 nm for Nile Red.

#### 2.8.2. Fourier Transform Infrared (FTIR) Spectrum Analysis

A VERTEX 70 spectrometer (Bruker, Bremen, Germany) was used to obtain the FTIR spectra. The freeze-dried gel samples were mixed with KBr and pressed into pellets. They were measured in the wavenumber range of 4000–400 cm^−1^ to obtain FTIR spectra, which were scanned 64× at a resolution of 4 cm^−1^.

#### 2.8.3. Scanning Electron Microscopy Analysis

All samples were immediately frozen with liquid nitrogen and then freeze-dried using a freeze dryer (FDU-2110, EYELA, Tokyo, Japan). The freeze-dried gel samples were gold-sputtered and observed under the Zeiss Merlin scanning electron microscope (Carl Zeiss, Oberkochen, Germany) at magnifications of 100× and 500× to examine the microstructure of the freeze-dried gel.

### 2.9. Statistical Analysis

All experiments were conducted in triplicate and the results were presented as mean ± standard deviation. The data was analyzed using SPSS 25 software, with statistical significance determined at a *p*-value of 0.05.

## 3. Results and Discussion

### 3.1. Effect of Peanut Oil Body Concentration on Particle Size and ζ-Potential of Mixed Gel Solutions

**Figure 1:**
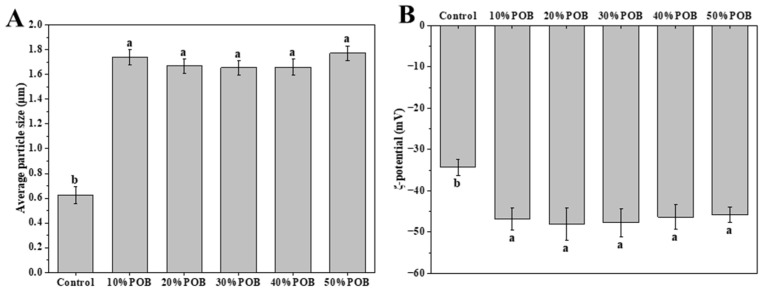
Average particle size (**A**) and ζ-potential (**B**) of the mixed gel solutions with different concentrations of OBs. Different letters indicate significant differences (*p* < 0.05).

We analyzed the mixed gel solutions prior to their gelation. As shown in Figure 1A, compared to the control solution without additional POBs, the particle size significantly increased. However, the gel solutions containing varying concentrations of POBs exhibited no significant differences. The ζ-potential serves as a crucial indicator of the stability of an emulsion system, reflecting the extent of the electrostatic attraction or repulsion between adjacent particles. The greater the absolute value of the potential, the higher the stability of the emulsion. After adding POBs, the absolute value of ζ-potential in the emulsion increased (Figure 1B). However, with an increase in the amount of added POBs, the absolute value of the potential remained essentially unchanged. This may be due to excess OBs flocculating in the continuous phase; previous studies demonstrated that a mixture of proteins and OBs can form a composite interface encompassed by protein clusters that has a tendency to interact and interfere [24]. Therefore, no statistically significant differences were observed in particle sizes and ζ-potentials between gel solutions with low and high concentrations of POBs. Liao et al. also found that adding a certain concentration range of oil bodies to the whey protein isolate solution did not significantly change the particle size and ζ-potential of the mixed gel solution [25].

### 3.2. Stability Analysis of Mixed Gel Solutions

**Figure 2:**
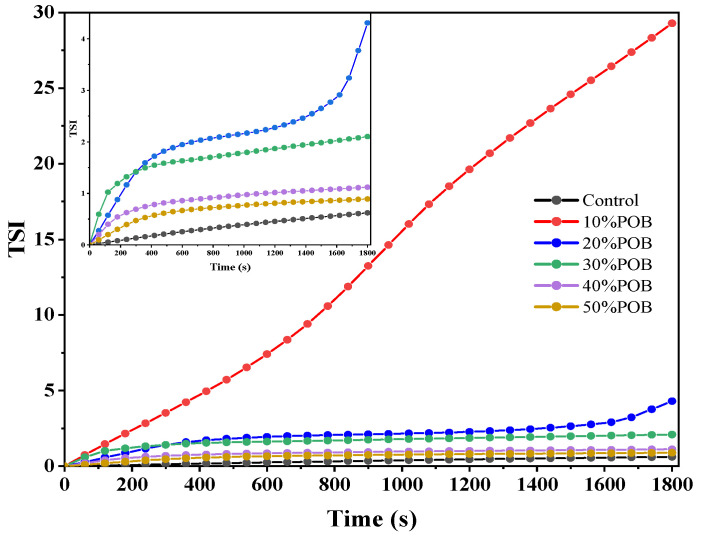
TSI values of OB emulsions with different OB concentrations.

The turbiscan stability index (TSI) characterizes the stability of emulsions. A smaller TSI indicates a limited range of backward scattering intensity changes, indicating higher stability [26]. The TSI values of the mixed gel solutions are depicted in Figure 2. As time progressed, the TSI values of all samples gradually increased, indicating a decreasing stability of the mixed solutions. In comparison to the control sample, the addition of 10% POBs caused a significant decrease in stability, resulting in a rapid change in the TSI value of the OB emulsion. However, with the continued increase of POBs added, the TSI value significantly decreased, leading to an enhanced stability of the OB emulsion. The TSI value at 50% POBs approached that of the control sample. After mixing with proteins, OBs form an interface surrounded by protein clusters, and interactions or interference between the interfaces can reduce the stability of the emulsion [24]. However, with an increased amount of POBs added, the stability of the OB emulsion improved. Similar results were drawn by Liu et al. [27], demonstrating that emulsions stabilized by OBs exhibited stronger stability at higher OB concentrations.

### 3.3. CLSM Microstructure Observations

**Figure 3:**
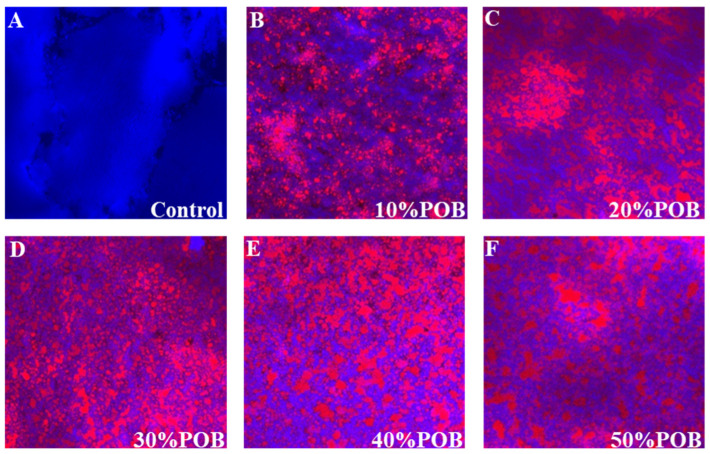
(**A**) CLSM images of the control, (**B**–**F**) CLSM images of the acid-induced OB emulsion-filled gel with different POB concentrations. Images depict the overlay of signals obtained from Nile red (fat, red coloured) and Nile blue (protein, blue coloured). Black denotes pores or void spaces.

The microstructure of the gel was characterized using CLSM. The gel prepared from SPI without POBs dispersion had a compact and dense gel network (Figure 3A). In contrast to the control, the addition of POBs altered the structure of the SPI gel induced by GDL. The discontinuous red fluorescence indicates that the OB emulsion-filled gel belonged to the oil-in-water type (Figure 3B–F). When the POB content was 10%, oil droplets dispersed in the SPI gel network (Figure 3B), and with increasing POBs content, the number of dispersed oil droplets increased. Oil droplet aggregation became more pronounced and the networks transformed from a protein network to a network of aggregated oil droplets (Figure 3C–F). POB aggregation formed larger oil droplets, hindering the formation of a compact and dense gel network, thus disrupting the gel structure [22].

### 3.4. SEM Microstructure Observations

**Figure 4:**
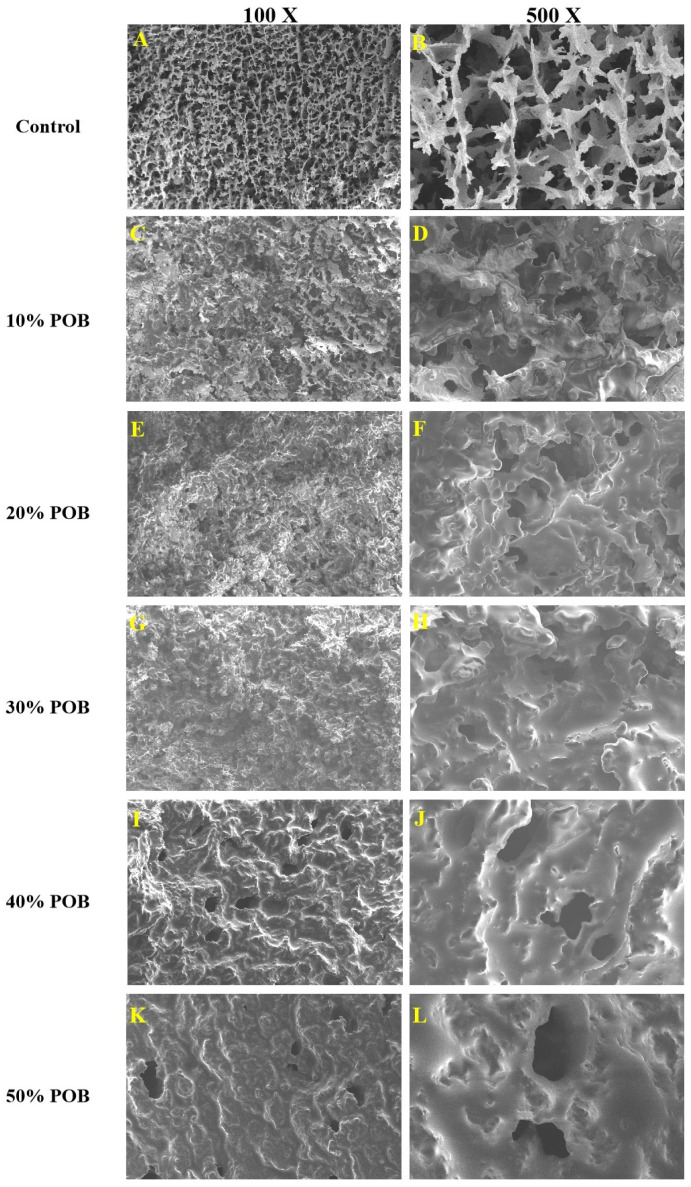
(**A**,**B**) SEM images of the control, (**C**–**L**) SEM images of the acid-induced OB emulsion-filled gel with different POB concentrations (10–50%). The images are magnified 100× and 500×, respectively.

The morphology of the OB emulsion-filled gel observed via SEM, see Figure 4, indicated that the POB content had a significant impact on the microstructure of the gel. In the absence of POBs, the surface and interior of the sample appear to form dense, uniform and interconnected pores, which created a compact network structure (Figure 4A,B). The addition of POBs to the SPI gel network increased the pore size, reduced density, and with increasing POBs content, the SPI gel was gradually covered by POBs (Figure 4C–L). The aggregation of oil droplets became more pronounced and the sample exhibited a smoother, undulating structure (Figure 4I–L). When the POB content exceeded 30%, due to the substantial aggregation of droplets during the acidification process, the gel contained flocculated particles and the particle gel was composed of a weakened protein network (Figure 4I,K). The gel network structure was weakened in this case.

### 3.5. FTIR Analysis

**Figure 5:**
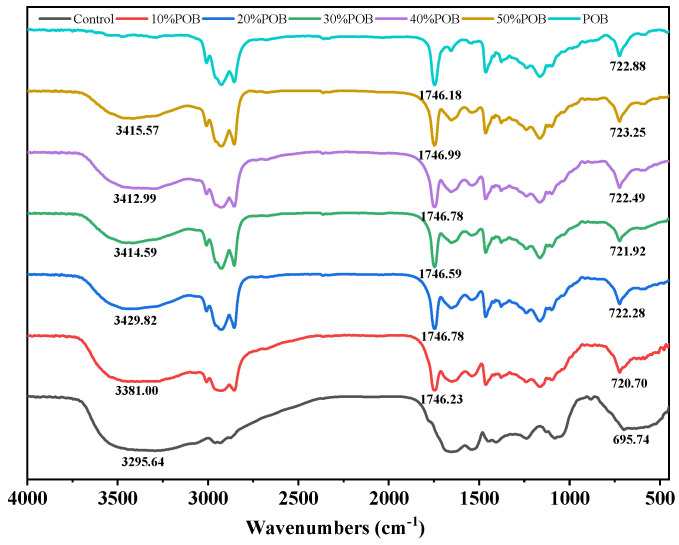
FTIR spectra of the lyophilized acid-induced OB emulsion-filled gel.

Different substances, due to their distinct molecular structures, absorb infrared radiation energy differently [28]. Therefore, FTIR spectroscopy can be employed to analyze potential interactions between SPI and POBs. The FTIR spectra showed similar peak patterns for different samples (Figure 5). The presence of intra- and inter-molecular hydrogen bonds was indicated by the occurrence of a wide absorption band in the range of 3200–3500 cm^−1^, which can be attributed to the stretching vibrations of -OH and -NH [29]. After the addition of POBs, the -OH stretching band of the OB emulsion-filled gel shifted to higher wavenumbers, indicating a strengthening of the hydrogen bonds. Meanwhile, asymmetric and symmetric stretching vibrations of -CH were observed at 2925 cm^−1^ and 2854 cm^−1^, possibly due to the oxidation of the POBs, forming -CH stretching vibrations in the saturated structure of the -CH3 and -CH2 groups. The stretching vibrations of triglycerides produced an absorption peak at 1745 cm^−1^ [30]. In comparison to the control group, the small absorption peak near 720 cm^−1^ in the SPI-POBs with added POBs represented alkyl -CH2 groups [31]. In contrast to the spectra of the SPI gel and the POBs, no new characteristic peaks appeared in the FTIR spectrum of the OB emulsion-filled gel. The absence of changes in functional groups suggests that no chemical reaction occurred between the SPI and POBs. The non-covalent interactions were the predominant interactions between the SPI and POBs.

### 3.6. Water Holding Capacity Analysis

**Figure 6:**
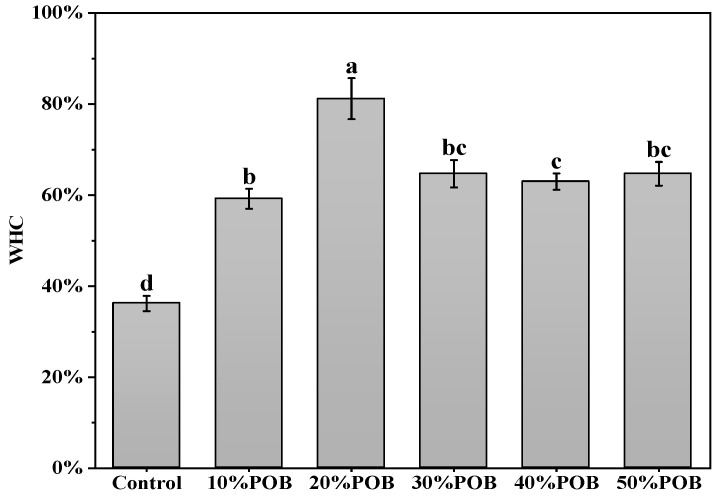
Water-holding capacity of the acid-induced OB emulsion-filled gel. Different letters indicate significant differences (*p* < 0.05).

The WHC represents the ability of the gel matrix to fix water molecules through capillary effects, which is one of crucial attributes in food [32]. The enhancement of gel strength and uniformity can improve water retention [33]. Gel filled with POBs had a better water-holding capacity (Figure 6). This was similar to previous research results by Li et al. [34]. The presence of a POB emulsion resulted in stronger capillary forces in the gel network, thereby improving the WHC. When the POB content was 20%, the gel exhibited its maximum WHC. However, as the POB content continued to increase to 30%, 40% and 50%, the WHC of the OB emulsion-filled gel significantly decreased. This was mainly due to the excess of OBs, leading to a reduction in the strength and density of the OB emulsion-filled gel, consequently reducing capillary forces. This made it difficult for the gel matrix to retain more water. Relevant findings were also documented by Liao et al. [25]. They found that an excess of OBs can lead to an enlargement of pores and a reduction in capillary forces in OB emulsion-filled gels, resulting in a decrease in the WHC.

### 3.7. Texture Profile Analysis

This section discusses the textural characteristics of the gel, which are important for consumer acceptance and preference in food. Hardness refers to the force required to affect a certain degree of deformation of raw food materials, while chewiness quantifies the energy necessary to masticate solid food into a swallowable state [35]. It can be observed from Table 1 that the addition of POBs significantly reduced the hardness of the OB emulsion-filled gel, which decreased with increasing amounts of POBs. Changes in chewiness were similar to those in hardness. The CLSM and SEM analyses indicated that the possible reason was that the addition of POBs hindered the formation of a gel network, reducing the gel density and strength. Hence, the hardness and chewiness of the gel decreased.

Springiness quantifies the extent of sample recovery during a compression cycle, cohesiveness represents the degree of deformation before the gel sample breaks, while gumminess is a secondary TPA parameter obtained by multiplying hardness and cohesiveness [36]. The table shows that the springiness of the OB emulsion-filled gel was significantly higher than that of the control sample. Gumminess decreased with the increase in POB content, while cohesiveness increased first and then decreased with the addition of POBs, but both were higher than in the control sample. The OB emulsion-filled gel exhibited characteristics similar to semi-solid foods with low hardness and high cohesiveness. The addition of POBs can enhance the springiness and cohesiveness of the SPI gel, but hardness, gumminess and chewiness will correspondingly decrease.

### 3.8. Rheological Properties

**Figure 7:**
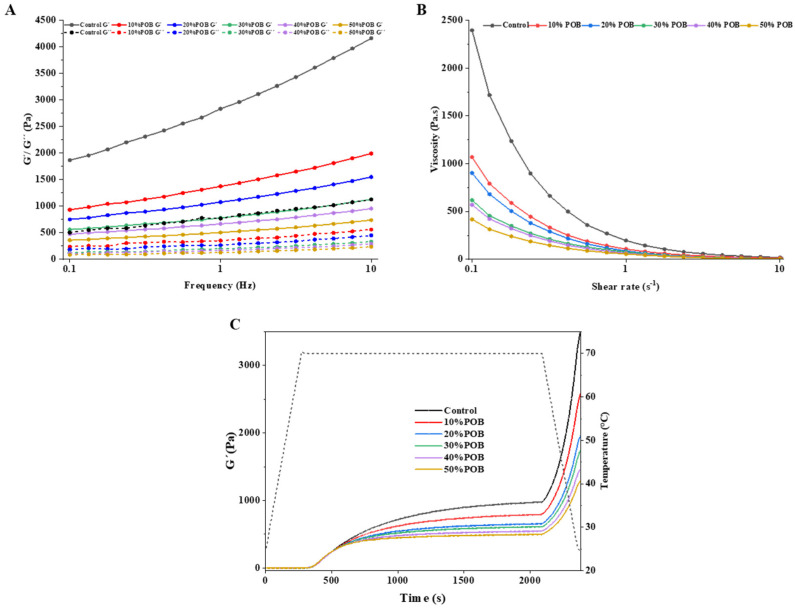
Rheological properties of the OB emulsion-filled gel with different POB concentrations. (**A**) Frequency-sweep curves with 0.1 to 10 Hz frequencies for the emulsion-filled gels. (**B**) Apparent viscosities of the OB emulsion-filled gel samples containing shear rates ranging from 0 to 10 s^−1^. (**C**) The gelation profile of the acid-induced OB emulsion-filled gels at various POB concentrations. The dashed line describes the temperature change during the measurement.

The storage modulus (G′) characterizes the energy retained in each dynamic oscillation cycle, reflecting the elasticity of the emulsion-filled gel, while the loss modulus (G″) represents the energy dissipation associated with the viscous (or liquid) characteristics [37]. As shown in Figure 7A, the storage and loss modulus of the acid-induced SPI gel filled with POBs gradually decreased with an increasing amount of POBs added. This indicated that as the amount of POBs added increased, the elasticity of the gel decreased and its network structure weakened. The storage modulus of all samples exhibited a significant increase with the increase in frequency, indicating the inherent frequency dependence of gel properties. The fact that the storage modulus was greater than the loss modulus for all samples suggests that all samples exhibited a viscoelastic solid behavior [38].

Based on the impact of oil droplets on gel characteristics, they can be categorized as active fillers or non-active fillers [1]. Active fillers bind or interact with the matrix, increasing the storage modulus of the emulsion-filled gel, while non-active fillers do not interact, or have only limited interactions, with the gel matrix, resulting in a decrease in the storage modulus [39]. The addition of active filler particles leads to an increase in the elastic modulus with the increase in volume fraction, whereas non-active fillers produce the opposite effect [40,41]. OBs can act as an active filler in gels constructed from proteins. Li et al. used soy protein isolate and flaxseed gum to construct two types of gels with different rice bran oil body (RBOB) concentrations through Ca^2+^ crosslinking, transglutaminase and Ca^2+^ double-crosslinking. RBOB acted as active filler in both types of emulsion-filled gels [34]. Sheikh et al. prepared mixed gels of gelatin and sesame protein with different OB contents through thermal induction, and found that OBs acted as strong and active fillers, enhancing the strength of composite gel structures [42].

In this study, increasing the POB content in the SPI gel gradually reduced the storage modulus, making the OB emulsion-filled gel softer compared to the SPI gel. Therefore, POBs played the role of non-active filler, which may be related to the method of inducing emulsion gelation. Nicole et al. studied the influence of different amounts of soybean oil on the rheological behavior of acid or salt-induced SPI gels, revealing a decrease in the storage modulus of acid-induced SPI gels with increasing volume fraction of soybean oil [43]. Gu et al. also found that soybean oil was a non-active filler in GDL-induced SPI emulsion gels, reducing the storage modulus [22]. The CLSM and SEM analysis results for the OB emulsion-filled gel indicated that after the GDL acidification of the OB emulsion, POBs aggregate at the isoelectric point, reducing their interactions with SPI. The aggregated POBs hindered the formation of a dense and strong gel network, resulting in a particulate gel composed of coagulated particles and a weakened protein network. 

All the gels exhibited a shear-thinning behavior (Figure 7B). This suggested the disruption of hydrogen bonds between proteins and protein particles, leading to a decreased gel strength [44]. At a constant shear rate, the OB emulsion-filled gel showed a gradual decrease in viscosity with an increasing amount of POBs. This was associated with the dispersion of POBs between protein networks, which acted as a non-active filler and formed a weak gel. This corroborated the conclusions reached from the hardness analysis.

The storage modulus can also be used to characterize the hardness of the gel [45]. Temperature sweeps recorded the changes of storage modulus during the formation of the OB emulsion-filled gel within 35 min (Figure 7C). The temperature gradually increased to 70 °C within the initial 5 min, while the gel hardness approached zero. After 5 min, the temperature was maintained at 70 °C for 30 min, and the hardness of the gel exhibited a time-dependent increase, with a gradual deceleration in the rate of increase. The increase in gel hardness is attributed to the hydrolysis of GDL, which lowers the pH to the SPI isoelectric point and the gel begins to form at that time. When GDL hydrolysis reaches the SPI isoelectric point, self-association and gelation of SPI occurred and the storage modulus of the gel gradually increased. Eventually, the peak was reached upon GDL hydrolysis [26]. During the cooling period from 70 °C to 25 °C, a sharp increase in gel hardness (storage modulus) was observed for all SPI gels, indicating further strengthening of the gel structure. This phenomenon could be ascribed to the formation of other non-covalent and covalent bonds within the gel structure, such as disulfide bonds and hydrogen bonds [46].

## 4. Conclusions

This study evaluated the impact of adding POBs on the characteristics of an SPI gel induced by GDL. The addition of POBs increased the WHC, springiness and cohesiveness of the SPI gel; however, the hardness, chewiness and storage and loss modulus decreased. The results obtained from SEM and CLSM analyses revealed that the incorporation of POB during the acidification process impeded the formation of compact gel, resulting in a weaken gel network structure and modified gel properties. Overall, POBs had a significant effect on the gelation characteristics of the acid-mediated SPI gel, which is of crucial importance for the application of POBs in acid-mediated SPI gels and the development of new SPI-gel products.

## Figures and Tables

**Table 1:** Textural analysis of the acid-induced OB emulsion-filled gel.

Sample	Hardness/g	Springiness	Cohesiveness	Gumminess/g	Chewiness/g
Control	101.60 ± 5.28 ^A^	0.89 ± 0.04 ^C^	0.27 ± 0.01 ^C^	27.13 ± 1.66 ^A^	24.17 ± 1.53 ^A^
10%POB	59.15 ± 1.62 ^B^	0.94 ± 0.015 ^A^	0.39 ± 0.01 ^A^	22.92 ± 0.9 ^B^	21.62 ± 0.95 ^B^
20%POB	43.86 ± 1.20 ^C^	0.94 ± 0.02 ^AB^	0.39 ± 0.01 ^A^	16.95 ± 0.52 ^C^	15.87 ± 0.66 ^C^
30%POB	41.68 ± 1.94 ^CD^	0.94 ± 0.02 ^AB^	0.36 ± 0.01 ^B^	14.97 ± 0.64 ^D^	14.03 ± 0.62 ^CD^
40%POB	39.66 ± 1.19 ^CD^	0.94 ± 0.02 ^A^	0.36 ± 0.01 ^B^	14.17 ± 0.08 ^DE^	13.32 ± 0.28 ^CD^
50%POB	37.62 ± 0.61 ^D^	0.90 ± 0.02 ^CB^	0.35 ± 0.01 ^B^	13.10 ± 0.46 ^E^	11.81 ± 0.55 ^D^

Note: Each value is the mean of three replicates, all measured values have been expressed as the mean ± standard deviation. Different letters indicate significant differences (*p* < 0.05).

## Data Availability

The original contributions presented in this study are included in the article; further inquiries can be directed to the corresponding authors.
